# Fibrotic Response of Human Trabecular Meshwork Cells to Transforming Growth Factor-Beta 3 and Autotaxin in Aqueous Humor

**DOI:** 10.3390/biom12091231

**Published:** 2022-09-03

**Authors:** Mengxuan Liu, Megumi Honjo, Reiko Yamagishi, Nozomi Igarashi, Natsuko Nakamura, Makoto Kurano, Yutaka Yatomi, Koji Igarashi, Makoto Aihara

**Affiliations:** 1 Department of Ophthalmology, Graduate School of Medicine, The University of Tokyo, Tokyo 113-8655, Japan; 2 Department of Ophthalmology, Tokyo Teishin Hospital, Tokyo 102-0071, Japan; 3 Division of Vision Research, National Institute of Sensory Organs, National Hospital Organization Tokyo Medical Center, Tokyo 152-8902, Japan; 4 Department of Clinical Laboratory Medicine, Graduate School of Medicine, The University of Tokyo, Tokyo 113-8655, Japan; 5 Department of Clinical Laboratory, The University of Tokyo Hospital, Tokyo 113-8655, Japan; 6 Bioscience Division, Reagent Development Department, AIA Research Group, TOSOH Corporation, Ayaseshi 252-1123, Japan

**Keywords:** human trabecular meshwork cells, transforming growth factor-beta 3, autotaxin

## Abstract

This study examines the potential role of transforming growth factor-beta 3 (TGF-β3) on the fibrotic response of cultured human trabecular meshwork (HTM) cells. The relationships and trans-signaling interactions between TGF-β3 and autotaxin (ATX) in HTM cells were also examined. The levels of TGF-β and ATX in the aqueous humor (AH) of patients were measured by an immunoenzymetric assay. The TGF-β3-induced expression of the fibrogenic markers, fibronectin, collagen type I alpha 1 chain, and alpha-smooth muscle actin, and ATX were examined by quantitative real-time PCR, Western blotting, and immunocytochemistry, and the trans-signaling regulatory effect of TGF-β3 on ATX expression was also evaluated. In HTM cells, the significant upregulation of ATX was induced by TGF-β3 at a concentration of 0.1 ng/mL, corresponding to the physiological concentration in the AH of patients with exfoliative glaucoma (XFG). However, higher concentrations of TGF-β3 significantly suppressed ATX expression. TGF-β3 regulated ATX transcription and signaling in HTM cells, inducing the upregulation of fibrogenic proteins in a dose-dependent manner. Trans-signaling of TGF-β3 regulated ATX transcription, protein expression, and signaling, and was thereby suggested to induce fibrosis of the trabecular meshwork. Modulation of trans-signaling between TGF-β3 and ATX may be key to elucidate the pathology of XFG, and for the development of novel treatment modalities.

## 1. Introduction

Glaucoma, which affects the optic nerve and causes progressive optic neuropathy, is one of the leading causes of blindness and irreversible vision loss worldwide [1,2,3]. Elevation of intraocular pressure (IOP) is considered to be the main risk factor for glaucoma, and reduction of IOP is the most effective method to prevent the development and progression thereof [4,5,6,7].

The trabecular meshwork (TM) plays a crucial role in maintaining IOP by balancing the production and outflow of aqueous humor (AH) in the human eye [8]. Fibrosis in the TM increases the resistance of the conventional AH outflow pathway in primary open-angle glaucoma (POAG), which is the most common type of glaucoma [9]. Remodeling and excess accumulation of extracellular matrix (ECM) materials, adhesive interactions, regulation of the contractile properties of TM cells, and decreased permeability have been reported in glaucoma, and degeneration of outflow pathway tissues can lead to TM dysfunction and AH outflow resistance, resulting in marked elevation of IOP [10,11,12]. Exfoliative glaucoma (XFG) is a severe type of glaucoma characterized by greater IOP fluctuation, visual field loss, and optic nerve damage compared to POAG [10,13]. Therefore, research is needed to determine the pathogenesis, progression, and mechanism of XFG, to identify potential therapeutic targets. Although abnormal accumulation of fibrillary ECM disrupts the drainage of the AH [10,11], the precise mechanisms underlying the pathological changes of the AH pathway in XFG are still unclear.

The transforming growth factor-beta (TGF-β) superfamily is a multifunctional family of cytokines, including three specific genes encoding three different TGF-β isoforms (TGF-β1–β3) [14]. The TGF-βs are known as profibrotic cytokines, which cause tissue fibrosis and regulate the synthesis of ECM proteins involved in fibrosis in different tissues [5,15,16,17]. In the human eye, the TGF-β family may play a role in glaucoma by regulating collagen synthesis and causing tissue fibrosis, which participates in fibrotic glaucomatous pathologies in the TM, resulting in IOP elevation [18] via both canonical and noncanonical pathways [19,20].

TGF-β2 is upregulated in patients with POAG, but downregulated in patients with XFG, while TGF-β3 is markedly elevated in the AH of XFG patients compared to those with other types of glaucoma [5,21,22,23]. Recently, we demonstrated that the aqueous concentrations of lysophosphatidic acid (LPA) and the LPA-generating enzyme autotaxin (ATX) were significantly correlated with IOP, and were higher in patients with secondary open-angle glaucoma and XFG [24]. LPA is a major bioactive lipid mediator of fibrosis, and enhances outflow resistance in the conventional pathway [25]. We also demonstrated that TGF-β2 trans-signaling potently regulates ATX transcription and signaling in the TM, which may regulate the pathogenesis of the different glaucoma subtypes [5].

Several studies have focused on evaluating the effects and mechanisms of action of TGF-β1 and TGF-β2 in human trabecular meshwork (HTM) cells [5,26,27], while less is known about TGF-β3. The modulatory effects of TGF-β3 on fibrosis in HTM cells, and the potential correlation with the pathogenesis of XFG, are unclear. This study was performed to investigate the role of TGF-β3 in fibrotic changes of HTM cells in vitro, and to determine possible mechanisms of the pathogenesis of XFG related to the ATX signaling pathway.

## 2. Materials and Methods

### 2.1. AH Samples from Patients

AH samples were obtained from patients undergoing cataract surgery or glaucoma surgery between March 2014 and December 2019 at the University of Tokyo Hospital and three affiliated eye clinics. This prospective observational study was approved by the Institutional Review Board of the University of Tokyo and registered with the University Hospital Medical Information Network Clinical Trials Registry of Japan (ID: UMIN000027137). All procedures conformed to the tenets of the Declaration of Helsinki. Each patient provided written informed consent. Patients with open-angle glaucoma were classified into POAG and XFG groups, as described previously [28]. For age matching with XFG patients, control subjects, and patients of normal tension glaucoma (NTG) and POAG with high IOP into POAG group that aged ≥ 20 years were included. Exclusion criteria included other types of glaucoma, such as primary angle-closure or congenital/developmental glaucoma, and a previous history of intraocular surgery other than small-incision cataract surgery without complications. When both eyes of a patient met the inclusion criteria, only the eye treated first was included in the analyses.

Preoperative AH was obtained at the start of surgery before any incisional procedures, using limbal paracentesis and a syringe with a 30-gauge needle. Approximately 70–100 μL of AH was collected into a PROTEOSAVE SS 1.5 mL Slimtube (Sumitomo Bakelite, Tokyo, Japan), registered, and stored at −80 °C until processing.

### 2.2. Measurement of ATX, ATX Isoforms, TGF-β1, TGF-β2, and TGF-β3 in the AH

AH samples were collected as described previously [29]. Levels of ATX in the AH were determined by a two-site immunoenzymatic assay with an ATX assay reagent using the AIA system (Tosoh, Tokyo, Japan). TGF-β levels in the AH were measured using a Bio-Plex Pro TGF-β assay kit (Bio-Rad Laboratories, Hercules, CA, USA) in accordance with the manufacturer’s protocol.

### 2.3. Cell Culture and Passage

Primary HTM cells were isolated from human donor eyes without glaucoma (46, 52, and 55 years old) and characterized as described previously [30], in accordance with the method of Keller et al. [31]. HTM cells purchased from ScienCell Research Laboratories (San Diego, CA, USA) were also characterized and used. Cells were cultured in Dulbecco’s modified Eagle’s medium (DMEM) containing 10% fetal bovine serum (FBS) and antibiotic-antimycotic solution (100×) (Sigma-Aldrich, St. Louis, MO, USA) at 37 °C and 5% CO_2_.

To examine the effects of TGF-β3 (Wako Pure Chemical Industries, Ltd., Osaka, Japan), confluent HTM cells from passages 3 to 6 were incubated in a serum-free medium for 24 h. The cells were then treated with different concentrations of TGF-β3 for 72 h, with or without adding Smad3 inhibitor (SIS3) at the same time. The medium was replaced with the serum-free medium for 24 h after exposure. In the control group, the medium was changed at the same time points but only the vehicle was added in place of TGF-β3 or SIS3. All experiments were performed at least three times, and consistency was confirmed using biological replicates.

### 2.4. WST-1 Assay

HTM cells viability was assessed using the WST-1 assay (Dojindo Co, Tokyo, Japan) according to the manufacturer’s protocol. HTM cells were cultured in 96-well plates overnight and then incubated in the serum-free medium for 24 h. The cells were treated with TGF-β3 under the concentration of 100 ng/mL for 24 h and 72 h, and the cells’ survival was measured using the multimode plate reader (PerkinElmer, Waltham, MA, USA).

### 2.5. Immunocytochemistry

Immunocytochemistry was performed as described previously [5,27]. The primary antibodies were anti-ATX (1:200; MBL, Nagoya, Japan), anti-collagen type I alpha 1 chain (COL1A1, 1:400; Rockland Immunochemicals, Limerick, PA, USA), anti-alpha-smooth muscle actin (α-SMA, 1:400; Dako, Agilent, Santa Clara, CA, USA), anti-fibronectin (1:300; Santa Cruz Biotechnology, Dallas, TX, USA), and rhodamine phalloidin (7:1000; Thermo Fisher Scientific, Waltham, MA, USA). Alexa Fluor 488- and Alexa Fluor 594-conjugated secondary antibodies (1:1000) were purchased from Thermo Fisher Scientific. Images were obtained using a BX51 fluorescence microscope (Olympus, Tokyo, Japan), and quantitative analyses of the immunocytochemistry were performed as described previously [29,32].

### 2.6. Real Time Quantitative Polymerase Chain Reaction (qPCR)

The cells were lysed using ISOGEN (Nippon Gene, Tokyo, Japan) and mRNA was isolated using chloroform and isopropyl alcohol. A PrimeScript RT Reagent Kit (Takara Bio, Shiga, Japan) was used to synthesize cDNA from mRNA after isolation. The mRNA levels were quantified using the ΔΔCt method, as described previously [33]. The sequences of primers purchased from Hokkaido System Science (Hokkaido, Japan) used in this study were taken from previously published reports. The primers used for PCR were as follows: COL1A1, forward, 5′-CAGCCGCTTCACCTACAGC-3′ and reverse, 5′-TTTTGTATTCAATCACTGTCTTGCC-3′; fibronectin, forward, 5′-AAACCAATTCTTGGAGCAGG-3′ and reverse, 5′-CCATAAAGGGCAACCAAGAG-3′; α-SMA, forward, 5′-CCGACCGAATGCAGAAGGA-3′ and reverse, 5′-ACAGAGTATTTGCGCTCCGAA-3′; connective tissue growth factor (CTGF), forward, 5′-CTCCTGCAGGCTAGAGAAGC-3′ and reverse, 5′-GATGCACTTTTTGCCCTTCTT-3′; ATX, forward, 5′-ACAACGAGGAGAGCTGCAAT-3′ and reverse 5′-AGAAGTCCAGGCTGGTGAGA-3′; TGF-β1, forward, 5′-CCCAGCATCTGCAAAGCTC-3′ and reverse 5′-GTCAATGTACAGCTGCCGCA-3′; TGF-β2, forward, 5′-TGCCGCCCTTCTTCCCCTC-3′ and reverse 5′-GGAGCACAAGCTGCCCACTGA-3′; TGF-β-induced protein, forward 5′-GTCCACAGCCATTGACCTTT-3′ and reverse 5′-GAGTTTCCAGGGTCTGTCCA-3′; and GAPDH, forward, 5′-AATTCCATGGCACCGTCAAG-3′ and reverse, 5′-ATCGCCCCACTTGATTTTGG-3′. The level of gene expression was normalized relative to GAPDH.

### 2.7. Western Blotting

Western blotting was performed as described previously [33]. Briefly, cell lysates were collected in RIPA buffer (Thermo Fisher Scientific) containing protease inhibitors (Roche Diagnostics, Basel, Switzerland) after treatment. Protein concentrations were determined by BCA Protein Assay Kit (Thermo Fisher Scientific) according to the manufacturer’s protocol using bovine serum albumin as a standard. Protein extracts were separated by SDS-PAGE and transferred to PVDF membranes (Bio-Rad Laboratories). Then, the membranes were immersed in primary antibodies overnight at 4 °C. The primary antibodies were anti-phospho-STAT3 (phosphorylation statuses of signal transducer and activator of transcription 3, Tyr 705) (1:1000; Cell Signaling Technology, Inc., Danvers, MA, USA), anti-STAT3 (79D7) (1:1000; Cell Signaling Technology, Inc.), anti-phospho-SAPK (stress-activated protein kinase)/JNK (Jun amino terminal kinase) (Thy 183/Tyr185) (1:1000; Cell Signaling Technology, Inc.), anti-SAPK/JNK (1:1000; Cell Signaling Technology, Inc.), and anti-β-tubulin (1:1000; Wako Pure Chemical Industries, Ltd., Osaka, Japan). After washing, the membranes were incubated with horseradish peroxidase-conjugated anti-mouse or anti-rabbit secondary antibody (1:2000–1:5000; Thermo Fisher Scientific) for 1 h at room temperature. The membranes were reacted with ECL substrate (Thermo Fisher Scientific) followed by ImageQuant LAS 4000 mini (GE Healthcare, Chicago, IL, USA) and the bands were quantified with ImageJ software (ver. 1.49, NIH, Bethesda, MD, USA).

### 2.8. Statistical Analysis

All statistical analyses were performed using SPSS version 22.0 (IBM Corp., Armonk, NY, USA). Student’s *t*-test and Fisher’s exact test were used to compare two groups. The Steel–Dwass test was carried out to compare multiple variables. A one-way ANOVA followed by Tukey’s post-hoc test was used for the comparison of data among experimental groups. The data are presented as mean ± standard error (SE) and mean ± standard deviation (SD). In all analyses, *p* < 0.05 was taken to indicate statistical significance.

## 3. Results

### 3.1. The Levels of ATX, TGF-β1, TGF-β2, and TGF-β3 in the AH of POAG and XFG Patients

A total of 95 eyes of 95 patients, consisting of 39 eyes without any ocular complications (control), 27 eyes with POAG, and 29 eyes with XFG, were included in the study. The demographic characteristics of the study population are listed in Table 1.

**Figure 1:**
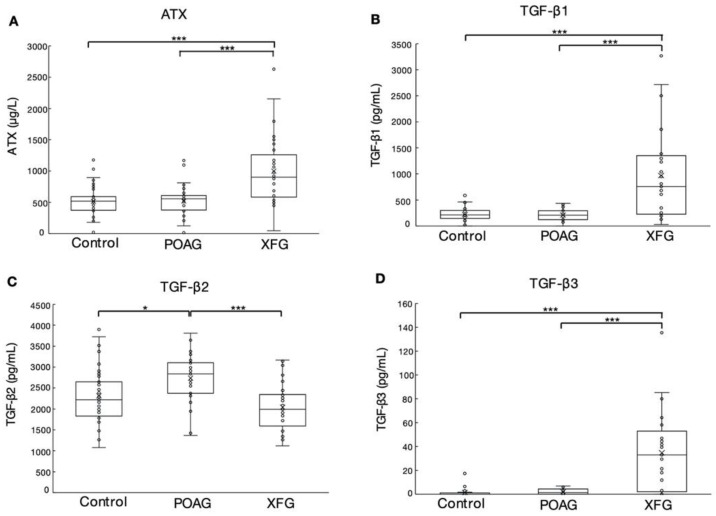
Levels of ATX, TGF-β1, TGF-β2, and TGF-β3 in the AH. (**A**) The level of ATX, as measured by immunoenzymetric assay was significantly higher in the XFG than control and POAG groups (both *p* < 0.001). (**B**) The level of TGF-β1 was significantly higher in the XFG than control and POAG groups (both *p* < 0.001). (**C**) The level of TGF-β2 showed the inverse tendency, especially between the POAG and XFG groups (*p* < 0.05 between control and POAG, *p* < 0.001 between POAG and XFG). (**D**) The level of TGF-β3 was significantly higher in the XFG than control and POAG groups (both *p* < 0.001). * *p* < 0.05, *** *p* < 0.001.

Aqueous ATX levels were significantly higher in the XFG than control and POAG groups (both *p* < 0.001, Figure 1A). TGF-β1 levels were significantly higher in the XFG group than the other groups (*p* < 0.001, Figure 1B). TGF-β2 levels were significantly higher in the POAG than control and XFG groups (*p* < 0.05 and *p* < 0.001, respectively, Figure 1C). TGF-β3 levels were significantly higher in the XFG group compared with the control and POAG groups (both *p* < 0.001, Figure 1D). The average TGF-β3 level in the XFG group was 34.7 ± 6.2 (SD), with a maximum value of 135.35 pg/mL.

### 3.2. Effects of TGF-β3 in HTM Cell Viability

We treated cultured HTM cells with 100 ng/mL of TGF-β3 to evaluate whether the high concentration of TGF-β3 cause cytotoxic to the cells. After a stimulation with 100 ng/mL TGF-β3 for 24 h, the results indicated that it did not induce significant changes in HTM cells viability. Also, stimulating with 100 ng/mL TGF-β3 for 72 h did not induce significant changes in cells viability compared to the control group (Supplementary Figure S1, *n* = 4).

### 3.3. Effects of TGF-β3 on COL1A1, CTGF, Fibronectin, and α-SMA in HTM Cells

**Figure 2:**
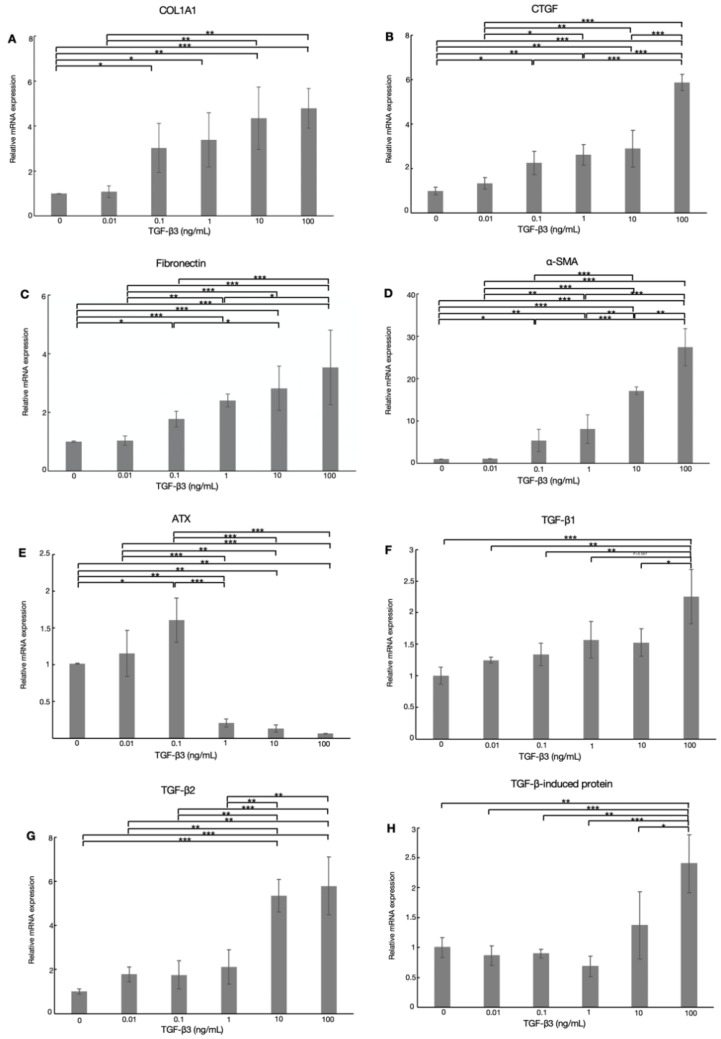
Real-time qPCR analysis of the mRNA levels of (**A**) COL1A1, (**B**) CTGF, (**C**) fibronectin, (**D**) α-SMA, (**E**) ATX, (**F**) TGF-β1, (**G**) TGF-β2, and (**H**) TGF-β-induced protein after stimulation with different concentrations (0, 0.01, 0.1, 1, 10, 100 ng/mL) of TGF-β3 in HTM cells. The mRNA expression levels of COL1A1, CTGF, fibronectin, α-SMA, TGF-β1, TGF-β2, and TGF-β-induced protein were significantly increased after treatment with TGF-β3. The ATX mRNA level was significantly increased with 0.1 ng/mL TGF-β3 treatment and significantly decreased with >1 ng/mL TGF-β3. GAPDH was used as an internal control for normalization. Data are presented as the mean ± standard deviation. * *p* < 0.05, ** *p* < 0.01, *** *p* < 0.001, *n* = 5.

We next investigated the effects of TGF-β3 on fibrogenic proteins in HTM cells. The levels of COL1A1, CTGF, fibronectin, and α-SMA mRNA were significantly upregulated on treatment with ≥0.1 ng/mL TGF-β3, in a dose-dependent manner (Figure 2A–D, *n* = 5). It was suggested that TGF-β3 with a physiological concentration as low as 0.1 ng/mL, which was comparable to the physiological AH concentration in the AH of XFG patients, can exert significant effects on fibrotic changes in HTM cells, although the effects were stronger with higher concentrations.

### 3.4. Effects of TGF-β3 on ATX, TGF-β1, TGF-β2, and TGF-β-Induced Protein in HTM Cells

We also investigated the dose-dependent effects of TGF-β3 on ATX, TGF-β1, TGF-β2, and TGF-β-induced protein expression in HTM cells by real-time qPCR. The results showed that the relative expression of ATX mRNA was significantly increased on treatment with 0.1 ng/mL TGF-β3, but notably decreased with 1, 10, or 100 ng/mL TGF-β3 compared to the control group (Figure 2E, *n* = 5). In comparison with the control group, the relative level of TGF-β2 expression increased with higher concentrations of 10 and 100 ng/mL TGF-β3, but there were no significant changes with concentrations < 10 ng/mL (Figure 2G, *n* = 5). TGF-β1 and TGF-β-induced protein expression was significantly elevated after TGF-β3 treatment at higher concentrations up to 100 ng/mL, but there was no significant upregulation with concentrations below 10 ng/mL (Figure 2F,H, *n* = 5).

### 3.5. Effects of TGF-β3 on the ATX-Related Transcription Factors via TGF-β Noncanonical Pathway in HTM Cells

As we found that the TGF-β3 influenced ATX expression in a dose-dependent manner, we next examined whether TGF-β3 influenced ATX expression through the noncanonical pathway. To investigate the transcriptional regulation of ATX in HTM cells, western blotting was performed to evaluate the levels of total (t)-STAT3 and phospho (p)-STAT3, as well as total (t)-SAPK/JNK and phospho (p)-SAPK/JNK, as these transcription factors regulate ATX expression [34].

**Figure 3:**
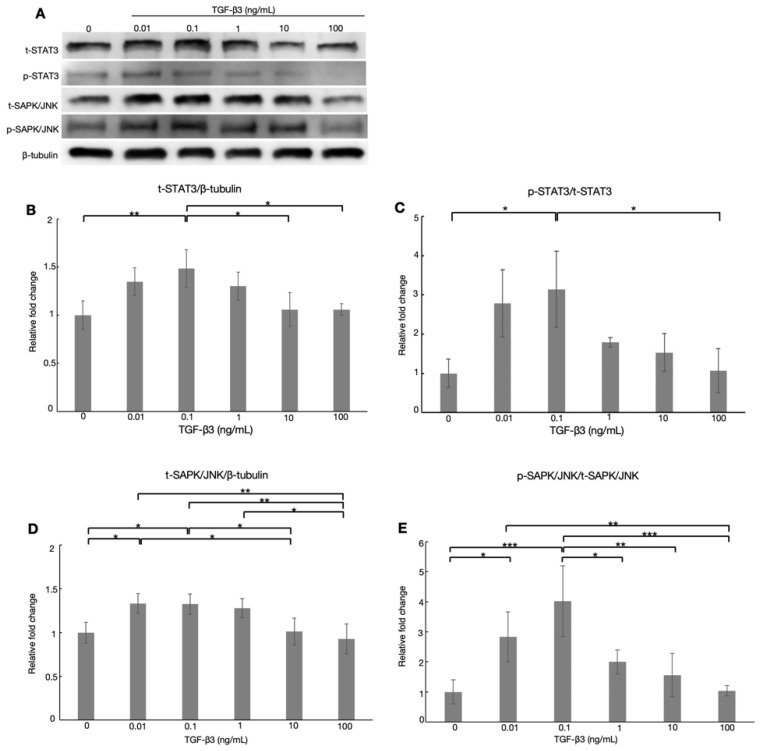
Western blotting of TGF-β3, statuses of signal transducer and activator of transcription 3 (STAT3), and stress-activated protein kinase (SAPK)/Jun amino terminal kinase (JNK) in HTM cells. Representative bands for STAT3 and SAPK/JNK are shown in (**A**). The total (t)-STAT3/β-tubulin ratio (**B**) and phosphorylated (p)-STAT3/t-STAT3 ratio (**C**) in HTM cells were significantly increased after treatment with 0.1 ng/mL TGF-β3 compared to controls, while the ratio was significantly decreased by treatment with 100 ng/mL TGF-β3 in comparison to 0.1 ng/mL TGF-β3. The t-SAPK/JNK/β-tubulin ratio (**D**) and p-SAPK/JNK/t-SAPK/JNK ratio (**E**) were significantly enhanced after treatment with 0.01 and 0.1 ng/mL TGF-β3 compared to controls. However, the ratio was significantly reduced by treatment with 10 and 100 ng/mL TGF-β3 in comparison to 0.1 ng/mL TGF-β3. The results are expressed relative to the loading control (β-tubulin). * *p* < 0.05, ** *p* < 0.01, *** *p* < 0.001, *n* = 5.

As shown in Figure 3, treatment with TGF-β3 at 0.1 ng/mL significantly increased t-STAT3 and p-STAT3 levels compared to the control group, while both were significantly lower in the 100 ng/mL TGF-β3 group compared to the 0.1 ng/mL TGF-β3 group (Figure 3B,C, *n* = 5). We also observed significant increases in t-SAPK/JNK and p-SAPK/JNK after treatment with 0.01 and 0.1 ng/mL TGF-β3 compared with the control group (Figure 3D,E, *n* = 5). In contrast, t-SAPK/JNK and p-SAPK/JNK were significantly decreased by treatment with 100 ng/mL TGF-β3 compared to 0.1 ng/mL TGF-β3. These results indicated that TGF-β3 supplementation increased the levels of total and phosphorylated STAT3 and SAPK/JNK at the lower concentration of 0.1 ng/mL, while they were reduced by the high concentration of 100 ng/mL in HTM cells.

### 3.6. Effects of TGF-β3 on the Expression of ATX via TGF-β Canonical Pathway in HTM Cells

As shown in Figure 3, the noncanonical TGF-β pathway may be involved in TGF-β3 concentration-dependent regulation of ATX expression. To determine whether TGF-β3 also regulates the changes in expression of ATX via the canonical TGF-β pathway in HTM cells, the Smad3 inhibitor SIS3 was used to evaluate the effects of Smad pathway inhibition on ATX expression by real-time qPCR and immunocytochemistry.

**Figure 4:**
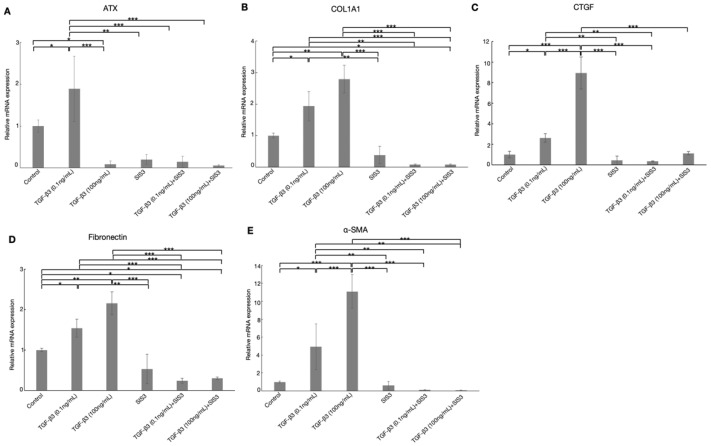
Real-time qPCR analysis of (**A**) ATX, (**B**) COL1A1, (**C**) CTGF, (**D**) fibronectin, and (**E**) α-SMA mRNA expression after treatment by TGF-β3 with or without the Smad3 inhibitor, SIS3, in HTM cells. The relative mRNA expression level of ATX was significantly upregulated by 0.1 ng/mL TGF-β3 and suppressed with SIS3 cotreatment. The level of ATX was significantly decreased by 100 ng/mL TGF-β3 treatment, but did not change after cotreatment with SIS3. The relative expression levels of COL1A1, CTGF, fibronectin, and α-SMA mRNA were significantly increased following treatment with 0.1 and 100 ng/mL TGF-β3 compared with the controls, but these enhancements were significantly decreased by cotreatment with SIS3. GAPDH was used as an internal control for normalization. Data are presented as the mean ± standard deviation. * *p* < 0.05, ** *p* < 0.01, *** *p* < 0.001, *n* = 5.

As shown in Figure 4A (*n* = 5), ATX mRNA expression was considerably increased after treatment with 0.1 ng/mL TGF-β3, but significantly downregulated after stimulation with TGF-β3 at a concentration of 100 ng/mL. The enhancement of ATX mRNA expression induced by 0.1 ng/mL TGF-β3 treatment was significantly attenuated by cotreatment with 10 μM SIS3. Treatment with 100 ng/mL TGF-β3, and cotreatment with TGF-β3 and SIS3, decreased the relative amounts of ATX to 0.09 ± 0.08 and 0.06 ± 0.02-fold, respectively, showing that the level of ATX was unaffected by SIS3 in the presence of a high concentration of TGF-β3. The suppression of ATX mRNA expression by 100 ng/mL TGF-β3 treatment was not attenuated by SIS3, suggesting that the enhancement of ATX expression is partly mediated by the canonical pathway, as well as the noncanonical pathway, while the effect of a low concentration of TGF-β3 is mainly mediated by the canonical pathway.

**Figure 5:**
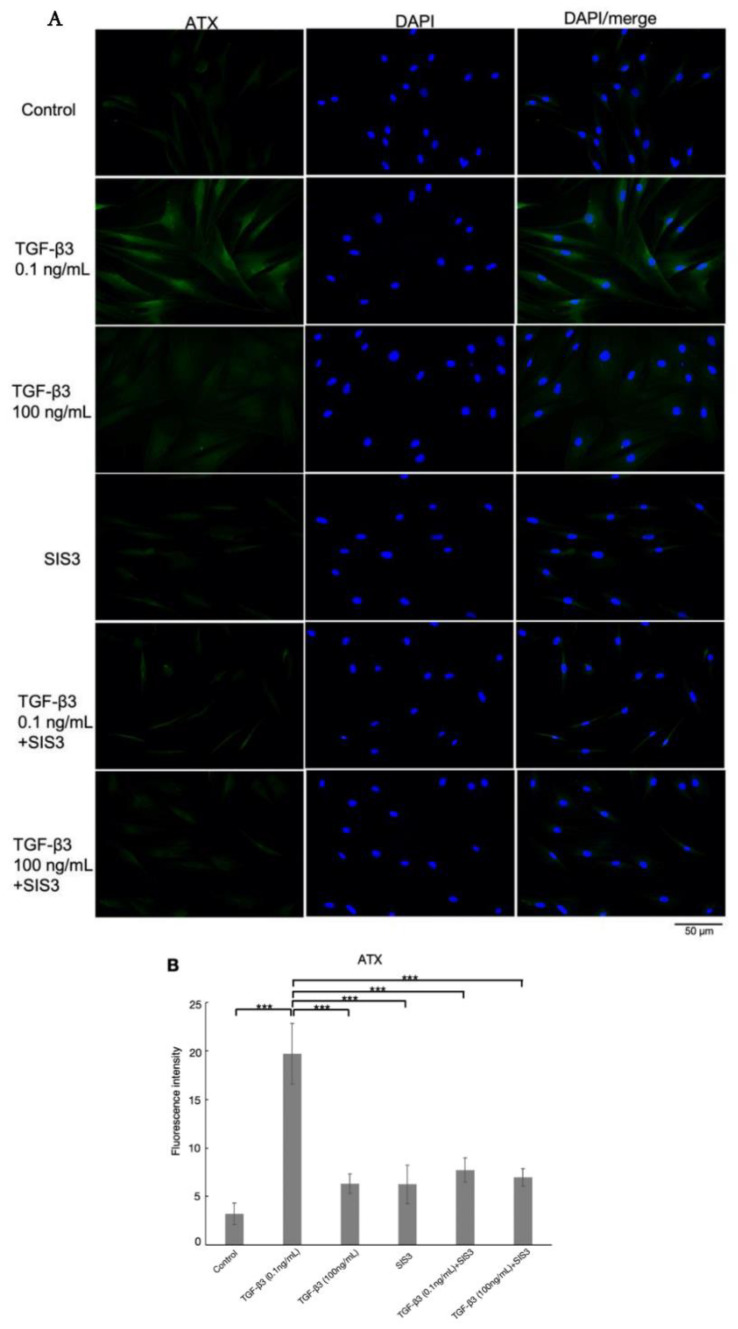
(**A**) Immunocytochemistry of ATX in HTM cells stimulated with TGF-β3, with or without the Smad3 inhibitor, SIS3, and (**B**) the quantitative results based on immunocytochemistry. The left panels show cells stained for ATX (green). The middle panels show cells stained with 4′,6-diamidino-2-phenylindole (DAPI, blue). The right panels show merged images. The expression of ATX was increased with 0.1 ng/mL TGF-β3 treatment, and the change was attenuated by SIS3 treatment significantly. Data are presented as the mean ± standard deviation. *** *p* < 0.001. Bar, 50 μm, *n* = 4.

Immunohistochemical analysis showed that the level of ATX protein expression was also significantly increased after treatment with 0.1 ng/mL TGF-β3 compared to the control group, but reduced after treatment with TGF-β3 at a concentration of 100 ng/mL compared to the 0.1 ng/mL TGF-β3 group significantly (Figure 5, *n* = 4). The upregulation of ATX protein expression was attenuated by cotreatment with SIS3 significantly.

### 3.7. TGF-β3 Affects Cytoskeletal Proteins and Fibrotic Changes via TGF-β Canonical Pathway in HTM Cells

To determine whether the TGF-β canonical pathway was involved in the cytoskeletal protein expression and fibrotic changes in HTM cells, we examined the effects of SIS3 and cotreatment with TGF-β3 by real-time qPCR and immunocytochemistry.

**Figure 6:**
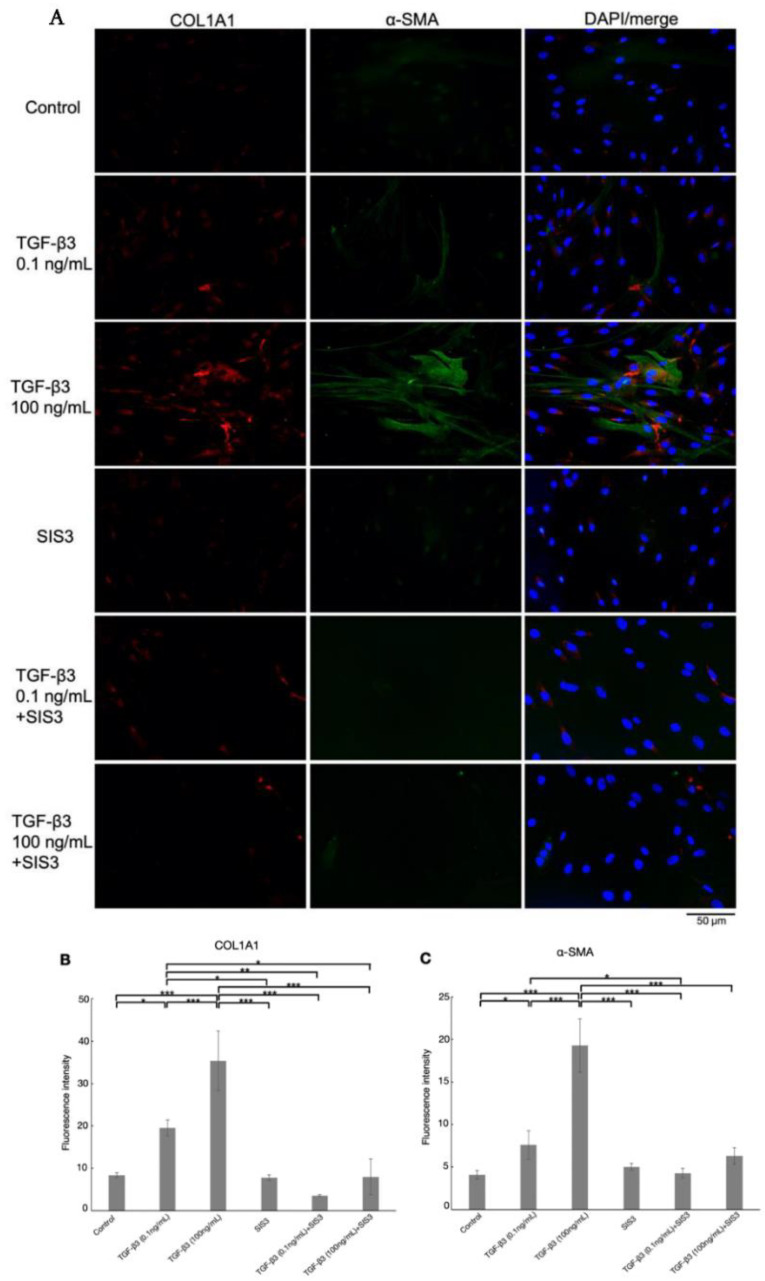
(**A**) Immunocytochemistry of COL1A1 and α-SMA in HTM cells treated with TGF-β3, with or without the Smad3 inhibitor, SIS3, and (**B**,**C**) the quantitative results based on immunocytochemistry. The left panels show cells stained for COL1A1 (red). The middle panels show cells stained for α-SMA (green). The right panels show merged images. The levels of COL1A1 and α-SMA expression were increased with 0.1 and 100 ng/mL TGF-β3 treatment, but the changes were suppressed by cotreatment with SIS3 significantly. Data are presented as the mean ± standard deviation. * *p* < 0.05, ** *p* < 0.01, *** *p* < 0.001. Bar, 50 μm, *n* = 4.

**Figure 7:**
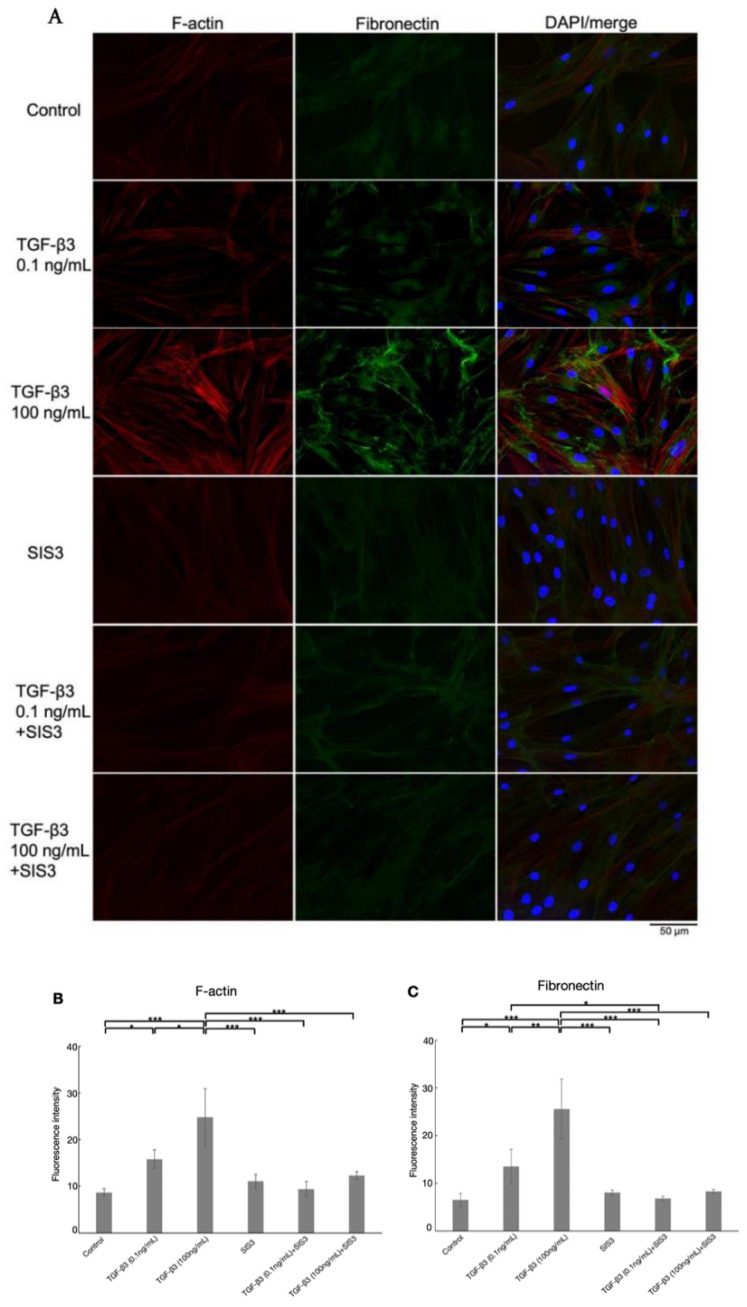
(**A**) Immunocytochemistry of F-actin and fibronectin in HTM cells after treatment with TGF-β3, with or without the Smad3 inhibitor, SIS3, and (**B**,**C**) the quantitative results based on immunocytochemistry. The left panels show cells stained for F-actin (red). The middle panels show cells stained for fibronectin (green). The right panels show merged images. The levels of F-actin were increased by 0.1 and 100 ng/mL TGF-β3 significantly, but the changes treated with 100 ng/mL TGF-β3 were suppressed by cotreatment with SIS3 significantly. The levels of fibronectin were significantly increased with 0.1 and 100 ng/mL TGF-β3, while the upregulated changes were reduced by cotreatment with SIS3 significantly. Data are presented as the mean ± standard deviation. * *p* < 0.05, ** *p* < 0.01, *** *p* < 0.001. Bar, 50 μm, *n* = 4.

As shown in Figure 6 and Figure 7, treatment with 0.1 and 100 ng/mL TGF-β3 significantly upregulated COL1A1, α-SMA, F-actin, and fibronectin protein expression in a dose-dependent manner. For protein expression of COL1A1, α-SMA, and fibronectin, upregulations were attenuated by cotreatment with SIS3 significantly. Additionally, cotreatment with SIS3 significantly decreased the protein expression of F-actin that was treated by 100 ng/mL TGF-β3. In addition, real-time qPCR showed that the levels of COL1A1, CTGF, fibronectin, and α-SMA mRNA were significantly increased by treatment with both 0.1 and 100 ng/mL TGF-β3 compared to the controls (Figure 4B–E, *n* = 5). Cotreatment with SIS3 significantly attenuated these changes. Taken together, these results suggest that the physiological concentration of TGF-β3 can induce a fibrotic reaction in HTM cells, at least partially via the canonical Smad3 pathway.

## 4. Discussion

In this study, we analyzed the effects of TGF-β3 on HTM cells, and found that TGF induced dose-dependent increases in the expression of COL1A1, CTGF, fibronectin, and α-SMA, while these effects were attenuated by the Smad3 inhibitor of SIS3 in HTM cells. In addition, TGF-β3 did not induce significant changes of HTM cells viability, but significantly upregulated TGF-β1, TGF-β2, and TGF-β-induced protein expression. The expression of ATX was also significantly increased by TGF-β3 treatment in HTM cells, especially by the lower concentration corresponding to the physiological level. This transactivation of ATX appeared to be mediated through the noncanonical STAT3 and SAPK/JNK pathway, as well as the canonical Smad3 pathway.

TGF-β2 has been suggested to play a critical role in the pathogenesis of POAG, based on its elevated levels in the AH of patients with POAG and its ability to induce ECM remodeling and TM fibrosis [35]. However, the molecular mechanisms involved in the generation of a glaucomatous environment in the various glaucoma subtypes remain unclear. Our previous in vitro and in vivo studies demonstrated that both TGF-β2 and ATX/LPA were involved in increased aqueous outflow resistance and IOP elevation, but the timing differed between these mediators, which may play specific roles in different glaucoma subtypes.

Several studies, including ours, investigated the effects and underlying mechanisms of TGF-β1 and TGF-β2 in HTM cells [5,26,27], but little is known about the action of TGF-β3 in HTM cells in vitro. XFG is a glaucoma subtype that causes more severe inflammation in comparison to the other subtypes of glaucoma, as well as sustained fibrosis and a poor prognosis [36]. We recently demonstrated that the levels of ATX and TGF-β3 in AH, which were strongly associated with IOP elevation, were markedly higher in patients with XFG than in controls and patients with other types of glaucoma [5,24]. We also reported that TGF-β3 and ATX in the AH showed good diagnostic performance in detecting glaucoma subtypes, and discriminated XFG from normal and POAG eyes; this indicated specific roles of TGF-β3 and ATX in the pathogenesis of XFG [24,28].

In the present study of AH in patients with glaucoma, the levels of TGF-β3 and ATX were significantly higher in XFG than the control group and POAG groups, whereas the level of TGF-β2 was downregulated in the AH in an inverse manner, consistent with results reported previously [24,28]. The concentration of TGF-β3 in the AH of XFG patients was extremely low compared to those of TGF-β1 and TGF-β2. Generally, the bioactive concentrations of TGF-βs in serum and tissue are higher than the amount in ng/mL [37]; however, it has also been reported that TGF-βs react with their receptors at lower concentrations of 0.5–20 pM [38]. Therefore, we first performed qPCR and investigated the effects of TGF-β3 at different concentrations on fibrotic changes in HTM cells. As shown in Figure 2, TGF-β3 induced fibrotic changes in a dose-dependent manner, which were especially marked at higher concentrations. However, we also confirmed that the lower physiological concentration of TGF-β3 was sufficient to promote fibrogenic reactions in HTM cells.

Next, we explored the effects of TGF-β3 on ATX, TGF-β1, TGF-β2, and TGF-β-induced protein expression. Notably, the mRNA and protein levels of ATX in HTM cells were significantly upregulated by exogenous TGF-β3 treatment at the lower concentration of  0.1 ng/mL, but significantly downregulated at a concentration of 1 ng/mL (Figure 2E and Figure 4A). In contrast, there were no significant changes in the expression of TGF-β1, TGF-β2, or TGF-β-induced protein with exogenous TGF-β3 treatment at concentrations below 10 ng/mL (Figure 2F–H). These findings highlight the differences in AH concentrations of TGF-β3 and ATX in XFG.

The levels of TGF-β1 and TGF-β3 in the AH showed similar tendencies to the ATX levels. TGF-β1 has also been suggested to play an important role in the pathogenesis of glaucoma [39,40], and higher levels of the TGF-β1 isoform occur most commonly in XFG [41]; however, it has been suggested that high IOP itself may induce the expression of activated TGF-β1 in TM cells [42]. In addition, we previously found that the levels of TGF-β1 in the AH were also high, similar to ATX, in secondary glaucoma patients with Posner-Schlossman syndrome, but there was no significant correlation between TGF-β1 and IOP [29]. Moreover, we showed that upregulation of ATX gene expression precedes TGF-β1 gene expression in our in vitro glaucoma model, suggesting that the induction of TGF-β1 expression is mediated by the ATX–LPA pathway via activation of the autocrine TGF-β1-Smad signaling pathway [29]. TGF-β1 may be at least partly involved in glaucomatous changes in the TM, but is unlikely to play a major role in the glaucomatous eye. Therefore, we focused on TGF-β3 to explore the specific role in HTM cells, and the relationship with ATX, in the present study.

TGF-βs induce fibrotic changes in HTM cells and other tissues by activating the noncanonical STAT3 and SAPK/JNK signaling pathways [5,20]. It was reported recently that the STAT3 and SAPK/JNK signaling pathways affect the expression of ATX [5,34]. Therefore, we next focused on the noncanonical pathways of STAT3 and SAPK/JNK, to analyze the crosstalk and relation between TGF-β3 and ATX and elucidate the mechanism underlying the pathogenesis of XFG. We used 0.1 and 100 ng/mL TGF-β3 to mimic physiological and high concentrations, respectively, and to modify the effective TGF-β3 levels in the AH, such that the mean TGF-β3 level in the AH was 34.67 ± 33.4 pg/mL, with a maximum of 135.4 pg/mL, in XFG (Figure 1).

As shown in Figure 3, t-STAT3, p-STAT3, t-SAPK/JNK, and p-SAPK/JNK were all significantly upregulated by treatment with 0.1 ng/mL TGF-β3, and downregulated by treatment with >0.1 ng/mL TGF-β3, compared to the 0.1 ng/mL TGF-β3 group. This concentration-dependent effect of TGF-β3 on STAT3 and SAPK/JNK expression showed a strong correlation with the ATX mRNA level, exhibiting a similar tendency to, and coinciding with the changes in, ATX expression. STAT3 is a direct transcriptional regulator of ATX, which is positively correlated with ATX expression [34,43]. In addition, we reported previously that the level of TGF-β3 was unaffected by ATX treatment in HTM cells [5], consistent with the present results suggesting that ATX is either a downstream mediator of, or is predominantly mediated by, TGF-β3. Therefore, we hypothesized that the concentration of ATX in the AH of XFG patients can be mediated by regulation of TGF-β3 through the STAT3 and SAPK/JNK signaling pathways. Further studies are needed to verify this hypothesis.

ATX was found to be a common constituent in the AH, and inhibition of ATX decreased the IOP in a rabbit model [44], indicating that it plays a crucial role in IOP regulation. ATX is a secreted enzyme that catalyzes the hydrolysis of lysophosphatidylcholine (LPC) to generate LPA [45]. LPA is a phospholipid mediator, the action of which is mediated by G protein-coupled receptors that evoke certain responses and induce actin stress fiber formation in almost all cell types, including HTM cells [44,46,47]. A number of studies have shown that the ATX–LPA signaling axis is involved in inflammation, wound healing, fibrosis, cancer, and metastasis [45,46,47,48,49]. In particular, the ATX–LPA pathway was shown to mediate fibrotic changes in HTM cells [28]. Dysregulation of the ATX–LPA pathway was shown to be involved in the pathogenesis of fibrosis via stimulation of Rho-mediated cytoskeletal remodeling, CTGF expression, cell contractility, and ECM deposition in HTM cells [44,50]. These observations were consistent with our finding that 0.1 ng/mL TGF-β3 significantly enhanced the expression of ATX, which may increase the levels of COL1A1, CTGF, fibronectin, and α-SMA in an autocrine or paracrine manner. However, as this was a shot-term in vitro study, further investigations will be needed to determine the effects of such trans-signaling between TGF-β3 and ATX on the fibrotic changes in HTM cells seen in glaucoma.

Interestingly, stimulation with 100 ng/mL TGF-β3 reduced the level of ATX, while COL1A1, CTGF, fibronectin, and α-SMA expression remained significantly elevated. To examine this phenomenon, we tested the effects of TGF-β3 on TGF-β1, TGF-β2, and TGF-β-induced protein expression, as several groups have demonstrated that TGF-β1 and TGF-β2 strongly upregulate the expression of CTGF and ECM in HTM cells through the canonical Smad3 pathway [5,16,20]. In addition, inhibition of TGF-β-induced protein expression increased TGF-β1 [51]. Our results showed that 0.1 ng/mL TGF-β3 had no effect on TGF-β1, TGF-β2, or TGF-β-induced protein expression in comparison to the control group. In contrast, the expression of TGF-β2 was significantly increased following treatment with ≥10 ng/mL TGF-β3, and TGF-β1 and TGF-β-induced protein expression was significantly upregulated by stimulation with 100 ng/mL TGF-β3 compared to controls. The upregulation of TGF-β2 mRNA expression was greater than that of TGF-β1 following treatment with 100 ng/mL TGF-β3, which may have been due to the increase in TGF-β-induced protein which reduced the level of TGF-β1. Therefore, we assumed that COL1A1, CTGF, fibronectin, and α-SMA were mainly upregulated by the effects of TGF-β2 instead of TGF-β1 when treated with 100 ng/mL TGF-β3.

To better understand the effects of TGF-β3 on ATX expression and ECM deposition, we further analyzed the TGF-βs canonical pathway using the Smad3 inhibitor, SIS3. The results of immunochemical analyses showed that treatment with 0.1 ng/mL TGF-β3 induced a greater increase in ATX, while cotreatment with SIS3 reduced this effect. Treatment with 0.1 and 100 ng/mL TGF-β3 induced changes in the distribution of F-actin and upregulated fibronectin, COL1A1, and α-SMA protein expression. These effects were attenuated by cotreatment with the SIS3. The COL1A1, CTGF, fibronectin, and α-SMA mRNA levels were upregulated by treatment with 0.1 and 100 ng/mL TGF-β3, while their expression was significantly attenuated following the addition of SIS3. Previous studies have shown that TGF-β3 has antifibrotic activity and plays an important role in inhibiting tissue fibrosis in human corneal cells and cardiac fibroblasts [18,52,53], while TGF-β3 significantly induced fibrotic changes in the present study. This discrepancy with previous reports may have been due to the lower concentrations and different cells used in the present study. The level of ATX mRNA expression was increased by 0.1 ng/mL TGF-β3 and significantly suppressed by treatment with SIS3. When stimulated with 100 ng/mL TGF-β3, the level of ATX was significantly reduced, but there were no significant differences after adding SIS3. This may have been because the trans-activation of TGF-β3 is mediated by both noncanonical signaling pathways and the canonical Smad pathway, where the noncanonical pathway may be predominantly responsible for the suppression of ATX expression induced by the treatment with the higher concentration of TGF-β3.

Several recent studies evaluated the roles of TGF-β1 and TGF-β2 in the pathogenesis of glaucoma using HTM cells [4,26,27]. Compared to the concentrations of TGF-β1 and TGF-β2, the level of TGF-β3 in the AH was low. However, the high level of TGF-β3 in patients with XFG suggests that TGF-β3 signaling may be an important therapeutic target for XFG, and we found that the physiological concentration of TGF-β3 can affect the fibrotic reaction in HTM cells and trans-signaling via the ATX–LPA pathway indeed. To the best of our knowledge, this is the first study to investigate the effects of TGF-β3 in HTM cells by acting on ATX, and the fibrotic changes in HTM cells appeared to involve the STAT3, SAPK/JNK, and Smad3 pathways. We also elucidated the potential mechanism and pathways involved in the pathogenesis of XFG, and hypothesized that the ATX–LPA–CTGF pathway may mediate the TGF-β3-induced cytoskeletal and fibrotic changes in HTM cells responsible for IOP elevation. We are aware of the limitation of our study that we observed the fibrogenic change of TM cells induced by the TGF-β3 at 72 h after stimulation, but it may be possible that the fibrogenic effect could be mediated by the trans-signaling and resultantly secreted other molecules. Furthermore, it may be also possible that the concentration-dependent reaction of TGF-β3 may be a common mechanism of action of these cytokines or a paradox effect triggered by unknown pathways. Further study will be needed to investigate the underlying mechanism between the alteration of TGF-β3 and ATX, or resultant cellular behavior.

**Figure 8:**
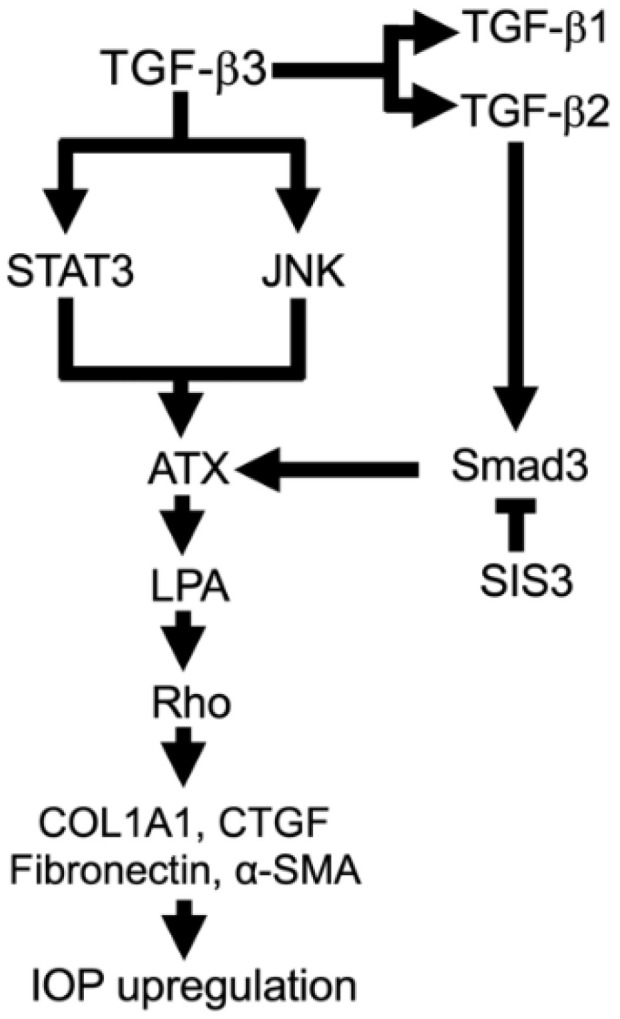
Potential mechanisms of TGF-β3-mediated ATX expression and fibrotic changes in HTM cells. ATX expression and fibrotic changes appeared to be partially mediated by the canonical pathway, as well as the noncanonical pathway.

XFG is a severe type of glaucoma causing greater IOP elevation, visual field loss, and optic nerve damage than other subtypes. However, there are still no effective treatments for this disease. Our study demonstrated the potential role of the TGF-β3–ATX–LPA–CTGF signaling pathway in the pathogenesis of XFG via regulation of the conventional outflow pathway (Figure 8). Although multiple complex physiological processes are likely involved in the pathology of XFG, inhibition of TGF-β3-ATX trans-signaling is a potential target to attenuate disease progression in patients with XFG.

## 5. Conclusions

Taken together, the results of the present study suggested that TGF-β3 may play a key role in modulating the biochemical composition of fibrosis and ECM deposition in HTM cells. Moreover, inhibition of TGF-β3-ATX trans-signaling in the TM may be critical to reduce resistance to the outflow facility of the TM and attenuate elevation of IOP in XFG.

## Figures and Tables

**Table 1:** Demographic characteristics of the study population.

Variables	Normal	POAG	XFG	*p* Value
Patients (n)	39	27	29	
Number of Eyes (n)	39	27	29	
Gender Ratio (male:female)	18:21	16:11	19:10	NS *
Ages (years)	77.1 ± 3.9	76.1 ± 3.9	75.5 ± 7.8	NS **
IOP (mmHg)	13.2 ± 3.2	16.4 ± 5.0	20.7 ± 7.7	†, †† **

The data are shown as the mean ± SD. * Fisher’s exact test. ** Steel–Dwass test. † *p* < 0.01 between control and POAG (Steel–Dwass test); †† *p* < 0.0001 between POAG and XFG (Steel–Dwass test). IOP, intraocular pressure; POAG, primary open-angle glaucoma; XFG, exfoliation glaucoma.

## Data Availability

All data reported are provided in the text or in the figures.

## Supplementary Materials

The following supporting information can be downloaded at: https://www.mdpi.com/article/10.3390/biom12091231/s1, Figure S1: HTM cell viability assay after stimulation with TGF-β3 at the concentration of 100 ng/mL for (A) 24 h and (B) 72 h.

## References

[1] Saito Y., Kizaki J., Wada Y., Shibasaki Y., Kishimoto N., Aihara M. (2021). Comparison of the 24-h Efficacy and Safety of Fixed Combination Carteolol/Latanoprost and Timolol/Latanoprost in Patients with Primary Open-Angle Glaucoma and Ocular Hypertension: A Prospective Crossover Study. Jpn. J. Ophthalmol..

[2] Omoto T., Fujishiro T., Asano-Shimizu K., Sugimoto K., Sakata R., Murata H., Asaoka R., Honjo M., Aihara M. (2020). Comparison of the Short-Term Effectiveness and Safety Profile of Ab Interno Combined Trabeculotomy Using 2 Types of Trabecular Hooks. Jpn. J. Ophthalmol..

[3] Fujita A., Sakata R., Ueda K., Nakajima K., Fujishiro T., Honjo M., Shirato S., Aihara M. (2021). Evaluation of Fornix-Based Trabeculectomy Outcomes in Japanese Glaucoma Patients Based on Concrete Long-Term Preoperative Data. Jpn. J. Ophthalmol..

[4] Montecchi-Palmer M., Bermudez J.Y., Webber H.C., Patel G.C., Clark A.F., Mao W. (2017). TGFβ2 Induces the Formation of Cross-Linked Actin Networks (CLANs) in Human Trabecular Meshwork Cells Through the Smad and Non-Smad Dependent Pathways. Investig. Ophthalmol. Vis. Sci..

[5] Igarashi N., Honjo M., Yamagishi R., Kurano M., Yatomi Y., Igarashi K., Kaburaki T., Aihara M. (2021). Crosstalk between Transforming Growth Factor β-2 and Autotaxin in Trabecular Meshwork and Different Subtypes of Glaucoma. J. Biomed. Sci..

[6] Wang J., Liu X., Zhong Y. (2013). Rho/Rho-Associated Kinase Pathway in Glaucoma. Int. J. Oncol..

[7] Aihara M., Ropo A., Lu F., Kawata H., Iwata A., Odani-Kawabata N., Shams N. (2020). Intraocular Pressure-Lowering Effect of Omidenepag Isopropyl in Latanoprost Non-/Low-Responder Patients with Primary Open-Angle Glaucoma or Ocular Hypertension: The FUJI Study. Jpn. J. Ophthalmol..

[8] Wang X., Huai G., Wang H., Liu Y., Qi P., Shi W., Peng J., Yang H., Deng S., Wang Y. (2017). Mutual Regulation of the Hippo/Wnt/LPA/TGF-β Signaling Pathways and Their Roles in Glaucoma (Review). Int. J. Mol. Med..

[9] Iyer P., Lalane R., Morris C., Challa P., Vann R., Rao P.V. (2012). Autotaxin-Lysophosphatidic Acid Axis Is a Novel Molecular Target for Lowering Intraocular Pressure. PLoS ONE.

[10] Angelilli A., Ritch R. (2009). Directed Therapy for Exfoliation Syndrome. TOOPHTJ.

[11] Raghunathan V.K., Morgan J.T., Chang Y.-R., Weber D., Phinney B., Murphy C.J., Russell P. (2015). Transforming Growth Factor Beta 3 Modifies Mechanics and Composition of Extracellular Matrix Deposited by Human Trabecular Meshwork Cells. ACS Biomater. Sci. Eng..

[12] Zhou Y., Xia X., Yang E., Wang Y., Marra K.G., Ethier C.R., Schuman J.S., Du Y. (2020). Adipose-derived Stem Cells Integrate into Trabecular Meshwork with Glaucoma Treatment Potential. FASEB J..

[13] Konstas A.G.P., Hollo G., Irkec M., Tsironi S., Durukan I., Goldenfeld M., Melamed S. (2007). Diurnal IOP Control with Bimatoprost versus Latanoprost in Exfoliative Glaucoma: A Crossover, Observer-Masked, Three-Centre Study. Br. J. Ophthalmol..

[14] Haque S., Morris J.C. (2017). Transforming Growth Factor-β: A Therapeutic Target for Cancer. Hum. Vaccines Immunother..

[15] Halder S.K., Goodwin J.S., Al-Hendy A. (2011). 1,25-Dihydroxyvitamin D3 Reduces TGF-Β3-Induced Fibrosis-Related Gene Expression in Human Uterine Leiomyoma Cells. J. Clin. Endocrinol. Metab..

[16] Massague J. (2000). Transcriptional Control by the TGF-Beta/Smad Signaling System. EMBO J..

[17] Zhang L., Liu X., Liang J., Wu J., Tan D., Hu W. (2020). Lefty-1 Inhibits Renal Epithelial–Mesenchymal Transition by Antagonizing the TGF-β/Smad Signaling Pathway. J. Mol. Hist..

[18] Karamichos D., Hutcheon A.E.K., Zieske J.D. (2011). Transforming Growth Factor-Β3 Regulates Assembly of a Non-Fibrotic Matrix in a 3D Corneal Model. J. Tissue Eng. Regen. Med..

[19] Zhang T., Wang X.-F., Wang Z.-C., Lou D., Fang Q.-Q., Hu Y.-Y., Zhao W.-Y., Zhang L.-Y., Wu L.-H., Tan W.-Q. (2020). Current Potential Therapeutic Strategies Targeting the TGF-β/Smad Signaling Pathway to Attenuate Keloid and Hypertrophic Scar Formation. Biomed. Pharmacother..

[20] Wilson S.E. (2021). TGF Beta −1, −2 and −3 in the Modulation of Fibrosis in the Cornea and Other Organs. Exp. Eye Res..

[21] Yoneda K., Nakano M., Mori K., Kinoshita S., Tashiro K. (2007). Disease-Related Quantitation of TGF-Beta3 in Human Aqueous Humor. Growth Factors.

[22] Garweg J.G., Zandi S., Gerhardt C., Pfister I.B. (2017). Isoforms of TGF-β in the Aqueous Humor of Patients with Pseudoexfoliation Syndrome and a Possible Association with the Long-Term Stability of the Capsular Bag after Cataract Surgery. Graefe’s Arch. Clin. Exp. Ophthalmol..

[23] Inatani M., Tanihara H., Katsuta H., Honjo M., Kido N., Honda Y. (2001). Transforming Growth Factor-Β2 Levels in Aqueous Humor of Glaucomatous Eyes. Graefe’s Arch. Clin. Exp. Ophthalmol..

[24] Igarashi N., Honjo M., Kurano M., Yatomi Y., Igarashi K., Kano K., Aoki J., Aihara M. (2018). Increased Aqueous Autotaxin and Lysophosphatidic Acid Levels Are Potential Prognostic Factors after Trabeculectomy in Different Types of Glaucoma. Sci. Rep..

[25] Rao P.V. (2014). Bioactive Lysophospholipids: Role in Regulation of Aqueous Humor Outflow and Intraocular Pressure in the Context of Pathobiology and Therapy of Glaucoma. J. Ocul. Pharmacol. Ther..

[26] Zhang Y., Tseng S.C.G., Zhu Y.-T. (2021). Suppression of TGF-Β1 Signaling by Matrigel via FAK Signaling in Cultured Human Trabecular Meshwork Cells. Sci. Rep..

[27] Nakamura N., Yamagishi R., Honjo M., Igarashi N., Shimizu S., Aihara M. (2021). Effects of Topical TGF-Β1, TGF-Β2, ATX, and LPA on IOP Elevation and Regulation of the Conventional Aqueous Humor Outflow Pathway. Mol. Vis..

[28] Honjo M., Igarashi N., Kurano M., Yatomi Y., Igarashi K., Kano K., Aoki J., Weinreb R.N., Aihara M. (2018). Autotaxin–Lysophosphatidic Acid Pathway in Intraocular Pressure Regulation and Glaucoma Subtypes. Investig. Ophthalmol. Vis. Sci..

[29] Igarashi N., Honjo M., Yamagishi R., Kurano M., Yatomi Y., Igarashi K., Kaburaki T., Aihara M. (2020). Involvement of Autotaxin in the Pathophysiology of Elevated Intraocular Pressure in Posner-Schlossman Syndrome. Sci. Rep..

[30] Igarashi N., Honjo M., Aihara M. (2021). MTOR Inhibitors Potentially Reduce TGF-Β2-Induced Fibrogenic Changes in Trabecular Meshwork Cells. Sci. Rep..

[31] Keller K.E., Bhattacharya S.K., Borrás T., Brunner T.M., Chansangpetch S., Clark A.F., Dismuke W.M., Du Y., Elliott M.H., Ethier C.R. (2018). Consensus Recommendations for Trabecular Meshwork Cell Isolation, Characterization and Culture. Exp. Eye Res..

[32] Jensen E.C. (2013). Quantitative Analysis of Histological Staining and Fluorescence Using ImageJ: Histological Staining/Fluorescence Using ImageJ. Anat. Rec..

[33] Honjo M., Igarashi N., Nishida J., Kurano M., Yatomi Y., Igarashi K., Kano K., Aoki J., Aihara M. (2018). Role of the Autotaxin-LPA Pathway in Dexamethasone-Induced Fibrotic Responses and Extracellular Matrix Production in Human Trabecular Meshwork Cells. Investig. Ophthalmol. Vis. Sci..

[34] Yang L., Yu X., Yang Y. (2018). Autotaxin Upregulated by STAT3 Activation Contributes to Invasion in Pancreatic Neuroendocrine Neoplasms. Endocr. Connect..

[35] Agarwal P., Daher A.M., Agarwal R. (2015). Aqueous Humor TGF-Β2 Levels in Patients with Open-Angle. Mol. Vis..

[36] Kottler U.B., Jünemann A.G.M., Aigner T., Zenkel M., Rummelt C., Schlötzer-Schrehardt U. (2005). Comparative Effects of TGF-Β1 and TGF-Β2 on Extracellular Matrix Production, Proliferation, Migration, and Collagen Contraction of Human Tenon’s Capsule Fibroblasts in Pseudoexfoliation and Primary Open-Angle Glaucoma. Exp. Eye Res..

[37] Khan S.A., Joyce J., Tsuda T. (2012). Quantification of Active and Total Transforming Growth Factor-β Levels in Serum and Solid Organ Tissues by Bioassay. BMC Res. Notes.

[38] Massague J. (1985). Transforming Growth Factor-/3 Modulates the High-Affinity Receptors for Epidermal Growth Factor and Transforming Growth Factor-c. 7. J. Cell Biol..

[39] Tamm E.R., Siegner A., Baur A., Lütjen-Drecoll E. (1996). Transforming Growth Factor-Β1 Induces α-Smooth Muscle-Actin Expression in Cultured Human and Monkey Trabecular Meshwork. Exp. Eye Res..

[40] Nakamura Y., Hirano S., Suzuki K., Seki K., Sagara T., Nishida T. (2002). Signaling Mechanism of TGF-β1–Induced Collagen Contraction Mediated by Bovine Trabecular Meshwork Cells. Investig. Ophthalmol. Vis. Sci..

[41] Schlötzer-Schrehardt U., Zenkel M., Küchle M., Sakai L.Y., Naumann G.O.H. (2001). Role of Transforming Growth Factor-Β1 and Its Latent Form Binding Protein in Pseudoexfoliation Syndrome. Exp. Eye Res..

[42] Kirwan R.P., Crean J.K., Fenerty C.H., Clark A.F., O’Brien C.J. (2004). Effect of Cyclical Mechanical Stretch and Exogenous Transforming Growth Factor-β1 on Matrix Metalloproteinase-2 Activity in Lamina Cribrosa Cells from the Human Optic Nerve Head. J. Glaucoma.

[43] Yemanyi F., Raghunathan V. (2020). Lysophosphatidic Acid and IL-6 Trans-Signaling Interact via YAP/TAZ and STAT3 Signaling Pathways in Human Trabecular Meshwork Cells. Investig. Ophthalmol. Vis. Sci..

[44] Ho L.T.Y., Osterwald A., Ruf I., Hunziker D., Mattei P., Challa P., Vann R., Ullmer C., Rao P.V. (2020). Role of the Autotaxin-Lysophosphatidic Acid Axis in Glaucoma, Aqueous Humor Drainage and Fibrogenic Activity. Biochim. Biophys. Acta (BBA)-Mol. Basis Dis..

[45] Tripathi H., Al-Darraji A., Abo-Aly M., Peng H., Shokri E., Chelvarajan L., Donahue R.R., Levitan B.M., Gao E., Hernandez G. (2020). Autotaxin Inhibition Reduces Cardiac Inflammation and Mitigates Adverse Cardiac Remodeling after Myocardial Infarction. J. Mol. Cell. Cardiol..

[46] Oikonomou N., Mouratis M.-A., Tzouvelekis A., Kaffe E., Valavanis C., Vilaras G., Karameris A., Prestwich G.D., Bouros D., Aidinis V. (2012). Pulmonary Autotaxin Expression Contributes to the Pathogenesis of Pulmonary Fibrosis. Am. J. Respir. Cell Mol. Biol..

[47] Trovato F.M., Zia R., Napoli S., Wolfer K., Huang X., Morgan P.E., Husbyn H., Elgosbi M., Lucangeli M., Miquel R. (2021). Dysregulation of the Lysophosphatidylcholine/Autotaxin/Lysophosphatidic Acid Axis in Acute-on-Chronic Liver Failure Is Associated With Mortality and Systemic Inflammation by Lysophosphatidic Acid–Dependent Monocyte Activation. Hepatology.

[48] Wang Z., Shi W., Tian D., Qin H., Vallance B.A., Yang H., Yu H.B., Yu Q. (2020). Autotaxin Stimulates LPA2 Receptor in Macrophages and Exacerbates Dextran Sulfate Sodium-Induced Acute Colitis. J. Mol. Med..

[49] Kostadinova L., Shive C.L., Anthony D.D. (2019). Elevated Autotaxin and LPA Levels during Chronic Viral Hepatitis and Hepatocellular Carcinoma Associate with Systemic Immune Activation. Cancers.

[50] Ninou I., Magkrioti C., Aidinis V. (2018). Autotaxin in Pathophysiology and Pulmonary Fibrosis. Front. Med..

[51] Bissey P.-A., Law J.H., Bruce J.P., Shi W., Renoult A., Chua M.L.K., Yip K.W., Liu F.-F. (2018). Dysregulation of the MiR-449b Target TGFBI Alters the TGFβ Pathway to Induce Cisplatin Resistance in Nasopharyngeal Carcinoma. Oncogenesis.

[52] Xue K., Zhang J., Li C., Li J., Wang C., Zhang Q., Chen X., Yu X., Sun L., Yu X. (2019). The Role and Mechanism of Transforming Growth Factor Beta 3 in Human Myocardial Infarction-induced Myocardial Fibrosis. J. Cell. Mol. Med..

[53] Karamichos D., Rich C.B., Zareian R., Hutcheon A.E.K., Ruberti J.W., Trinkaus-Randall V., Zieske J.D. (2013). TGF-Β3 Stimulates Stromal Matrix Assembly by Human Corneal Keratocyte-Like Cells. Investig. Ophthalmol. Vis. Sci..

