# Supplementary material for: Fibrotic Response of Human Trabecular Meshwork Cells to Transforming Growth Factor-Beta 3 and Autotaxin in Aqueous Humor
Source: Biomolecules. 2022 Sep 3;12(9):1231. doi: 10.3390/biom12091231 (PMC9496180; doi:10.3390/biom12091231)
Supplement: Supplementary file 1 [file biomolecules-12-01231-s001.zip › biomolecules-1877682-supplementary.pdf]

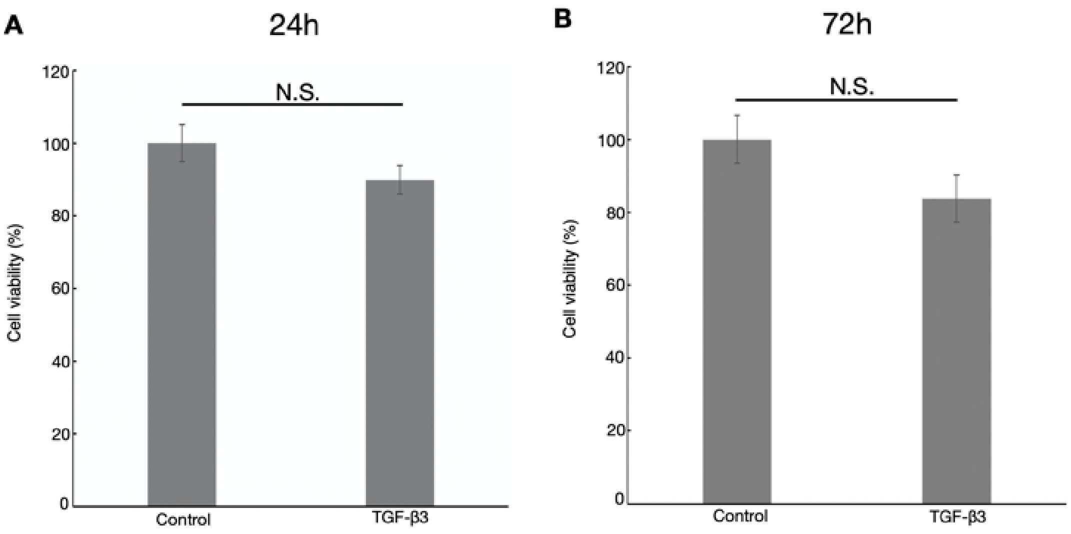

Supplementary Figure S1. HTM cell viability assay after stimulation with TGF-β3 at the concentration of 100 ng/mL for (**A**) 24 h and (**B**) 72 h. The results showed that treated with 100 ng/mL TGF-β3 for 24 h did not induce significant changes in HTM cells viability. Stimulation of 100 ng/mL TGF-β3 for 72 h did not induce significant changes in cells viability compared to the control group. Data are presented as the mean ± standard deviation, $n = 4$.
